# Acknowledgement of manuscript reviewers, the underappreciated contributors

**DOI:** 10.1186/1743-7075-12-3

**Published:** 2015-02-04

**Authors:** Yasmin Mulla, Ahmed Bakillah, M Mahmood Hussain

**Affiliations:** Editor-in-Chief, Nutrition & Metabolism, Kragujevac, USA

## Abstract

We and the Editorial Board sincerely acknowledge and thank all the reviewers for their active participation and contribution during 2014 (Volume 11). We greatly appreciate their dedication and behind the scenes contribution. It is largely due to their support and expertise that we have been able to publish high standard manuscripts. We would also like to thank authors for choosing Nutrition & Metabolism and contributing their cherished work to this journal.

**Sarah Abou Merhi**

United States of America

**Nader G. Abraham**

United States of America

**Charles Abramson**

United States of America

**Abdelaziz Abulelsaad**

Egypt

**Nihal Ahmad**

United States of America

**Kamal Amin**

Egypt

**Robin Anderson**

United States of America

**Susan Arthur**

United States of America

**Stella Aslibekyan**

United States of America

**Ramy Attia**

United States of America

**Maurizio Averna**

Italy

**Salman Azhar**

United States of America

**Ahmed Bakillah**

United States of America

**Michiel Balvers**

Netherlands

**Tyler Barker**

United States of America

**Darius Batulevicius**

Lithuania

**Jamie Baum**

United States of America

**Juerg H Beer**

Switzerland

**Elizabeth Beierle**

United States of America

**Damien Belobrajdic**

Australia

**Martha Belury**

United States of America

**Melissa Benton**

United States of America

**M'Hamed Bentourkia**

Canada

**Elizabeth Bertone-Johnson**

United States of America

**Deepak Bhatnagar**

United Kingdom

**Sheng Bi**

United States of America

**Jeffrey Billheimer**

United States of America

**William Blaner**

United States of America

**Emanuela Bostjancic**

Slovenia

**Olivier Bruyère**

Belgium

**Martin Burtscher**

Austria

**Andrew Butler**

United States of America

**Monika Cahova**

Czech Republic

**Edward Calabrese**

United States of America

**Philip Calder**

United Kingdom

**Matthew Cannon**

United States of America

**Luciana Caperuto**

Brazil

**Annalisa Castagna**

Italy

**Rolando Ceddia**

Canada

**Lisa Ceglia**

United States of America

**Giovanna Cenacchi**

Italy

**Xueying Chen**

United States of America

**Dong Cheng**

United States of America

**Mi-La Cho**

Korea, South

**Robert Coker**

United States of America

**Ian Cook**

South Africa

**Emmanuel Cosson**

France

**Philippe Costet**

United States of America

**Giuseppe Crescenzi**

Italy

**Stephen Cunnane**

Canada

**Alexandre Da Silveira**

Brazil

**Dominic D'Agostino**

United States of America

**Kezhi Dai**

United States of America

**Mark Daly**

United Kingdom

**Erick De Oliveira**

Brazil

**Nicole De Wit**

Netherlands

**Tamás Decsi**

Hungary

**Hans Degens**

United Kingdom

**Christos Derdemezis**

Greece

**Latha Devi**

United States of America

**Wen-Xing Ding**

United States of America

**Mohamed Dkhil**

Saudi Arabia

**Lharbi Dridi**

Canada

**Slavik Dushenkov**

United States of America

**Michael Eades**

United States of America

**Peter Eck**

Canada

**Abdalla El-Mowafy**

Egypt

**Henk Everts**

Netherlands

**Richard Feinman**

United States of America

**Arny Ferrando**

United States of America

**Eduardo Ferriolli**

Brazil

**Yvonne Fierz**

Switzerland

**Carmelo Fiore**

Italy

**Pavel Flachs**

Czech Republic

**James Fleet**

United States of America

**Leng Huat Foo**

Malaysia

**Ju Yen Fu**

Malaysia

**Jose Fuentes**

Spain

**Mary C. Gannon**

United States of America

**Mingming Gao**

United States of America

**Diego Garcia-Diaz**

Chile

**Eduardo Garcia-Fuentes**

Spain

**Amalia Gastaldelli**

Italy

**Abhisek Ghosal**

United States of America

**Barbara Gower**

United States of America

**Olaf Grisk**

Germany

**Romain Harmancey**

United States of America

**Robert Harris**

United States of America

**Susan Harris**

United States of America

**Morey Haymond**

United States of America

**Gerwin Heller**

Austria

**Darren Henstridge**

Australia

**Chi-Tang Ho**

United States of America

**Conrad Hodgkinson**

United States of America

**Adenilda Honorio-França**

Brazil

**Mary Honors**

United States of America

**Philip Hooper**

United States of America

**Chao-Tien Hsu**

Taiwan

**Yu Huang**

China

**Juha Hulmi**

Finland

**Jahangir Iqbal**

United States of America

**Eui-Bae Jeung**

Korea, South

**Xian-Cheng Jiang**

United States of America

**Mirim Jin**

Korea, South

**Julie Jurenka**

United States of America

**Douglas Kalman**

United States of America

**Konstantin Kandror**

United States of America

**Tadashi Kawai**

Japan

**Daniel Konrad**

Switzerland

**Mark Kotowicz**

Australia

**Chao-Qiang Lai**

United States of America

**Marcia Latorraca**

Brazil

**Donald Layman**

United States of America

**Mélanie Lemire**

Canada

**Tung Ming Leung**

United States of America

**Nate Lewis**

United States of America

**Ke Li**

United States of America

**Rui Li**

United States of America

**Qiang Li**

United States of America

**Fabio Lima**

Brazil

**Pao-Hwa Lin**

United States of America

**I-Min Liu**

Taiwan

**George Liu**

China

**Kiatlyn Liu**

United States of America

**Agnieszka Loboda**

Poland

**Jose Lopez-Miranda**

Spain

**Emil Lou**

United States of America

**Guojun Lu**

United States of America

**Bing Luan**

United States of America

**Marco Manca**

Netherlands

**Gaowei Mao**

United States of America

**Maria Raquel Marçal Natali**

Brazil

**M. Carmen Martinez**

France

**Vera Mazurak**

Canada

**Bodo Melnik**

Germany

**Ali Metwalli**

Saudi Arabia

**Christine Miaskowski**

United States of America

**Luca Miele**

Italy

**Mohammad Minhajuddin**

United States of America

**Bettina Mittendorfer**

United States of America

**Mahmoud Mohammad**

United States of America

**Srinidi Mohan**

United States of America

**Lesley Moisey**

Canada

**Maria Murholm**

Denmark

**Elza Muscelli**

Brazil

**Thirupathi Muthusamy**

United States of America

**Fernando Naclerio**

United Kingdom

**Takafumu Nagatomo**

Japan

**Joseph Nickels**

United States of America

**Estela Maria Novak**

Brazil

**Toshikatsu Okumura**

Japan

**Rytas Ostrauskas**

Lithuania

**Alok Pachori**

United States of America

**Xiaoyue Pan**

United States of America

**Athanasios G. Papavassiliou**

Greece

**Sandra Peake**

Australia

**Michael Pearen**

Australia

**Sandra Peters**

Canada

**Stephen Phinney**

United States of America

**Hanno Pijl**

Netherlands

**Earl Prinsloo**

South Africa

**Spencer Proctor**

Canada

**Denis Prud'Homme**

Canada

**Radhakrishna Rao**

United States of America

**Angela Resende**

Brazil

**Jong Rho**

Canada

**Denis Richard**

Canada

**Gerald Rimbach**

Germany

**Roberto Rivabene**

Italy

**Catarina Rodrigues Dos Santos**

Portugal

**Jean-Max Rouanet**

France

**Helene Rundqvist**

Sweden

**Arkady Rutkovskiy**

Norway

**Jennifer Ryan**

United Kingdom

**Fermin Sanchez De Medina**

Spain

**Sushma Sharma**

United States of America

**Fareeba Sheedfar**

Netherlands

**Laura Shelton**

United States of America

**Sangeetha Shyam**

Malaysia

**Adrian Slee**

United Kingdom

**James Soh**

United States of America

**Evelien Sohl**

Netherlands

**Guisheng Song**

United States of America

**Carol Souza Da Silva**

Netherlands

**Shanthi Srinivasan**

United States of America

**Alan Steel**

United Kingdom

**Barbara Strasser**

Austria

**Anne Sumner**

United States of America

**Guang Sun**

Canada

**Cenk Suphioglu**

Australia

**Arthur Swislocki**

United States of America

**Toshiaki Tamaki**

Japan

**Daniel Teitelbaum**

United States of America

**Inge Tetens**

Denmark

**Marie Thearle**

United States of America

**Min Tian**

United States of America

**Uwe Tietge**

Netherlands

**Angela Ting**

United States of America

**Janet Tou**

United States of America

**Robbert Touwslager**

Netherlands

**Ulrike Trendelenburg**

United Kingdom

**Tsuyoshi Tsuduki**

Japan

**Mariko Uehara**

Japan

**Angela Valverde**

Spain

**Evert Van Schothorst**

Netherlands

**Vasundara Venkateswaran**

Canada

**Adriana C Vidal**

United States of America

**Meghan Walsh**

United States of America

**Wan Zurinah Wan Ngah**

Malaysia

**Doug Ward**

United Kingdom

**Hope Weiler**

Canada

**Klaas Westerterp**

Netherlands

**Margriet Westerterp-Plantenga**

Netherlands

**Wanwarang Wongcharoen**

Thailand

**Zengying Wu**

United States of America

**Min Xia**

China

**Xunde Xian**

United States of America

**Feifei Xiao**

United States of America

**Yuquan Xiong**

United States of America

**Kazumasa Yamagishi**

United States of America

**Hua Yang**

China

**Kaiping Yang**

Canada

**Qinglin Yang**

China

**Zhong Ye**

United States of America

**Masanori Yoshizumi**

Japan

**Kai Yuan**

United States of America

**Fouad Zakharia**

United States of America

**Xiaodong Zhang**

United States of America

**Jinzhong Zhang**

United States of America

